# Supplementary material for: Intestinal microbiome dysbiosis increases Mycobacteria pulmonary colonization in mice by regulating the Nos2-associated pathways
Source: eLife. 2024 Oct 16;13:RP99282. doi: 10.7554/eLife.99282 (PMC11483126; doi:10.7554/eLife.99282)
Supplement: Supplementary file 1. — (a) The relative abundance of bacteria in CL and CON groups at phylum levels. (b) The relative abundance of bacteria in CL and CON groups at general level. (c) The relative abundance of fungi in CL and CON groups at the phylum level. (d) The relative abundance of fungi in CL and CON groups at the genera level. The Wilcoxon rank-sum test was performed between the two groups. CL: CL treatment group, CON: control group. [file elife-99282-supp1.docx]

**Supplementary file 1.** **The relative abundance of gut bacteria and fungi at the phylum and genera levels.**

**Supplementary file 1a. The relative abundance of bacteria in CL and CON groups at phylum levels**

| Species name | CL-mean(%) | CON-mean(%) | P_value |
| --- | --- | --- | --- |
| *p__Firmicutes* | 49.83 | 32.37 | 0.003876 |
| *p__Bacteroidota* | 27.08 | 51.25 | 0.000939 |
| *p__Proteobacteria* | 14.78 | 4.418 | 0.01008 |
| *p__Campylobacterota* | 7.648 | 4.838 | 0.8748 |
| *p__Verrucomicrobiota* | 0.007135 | 2.867 | 0.000682 |
| *p__Actinobacteriota* | 0 | 2.461 | 0.00041 |
| *p__Deferribacterota* | 0 | 1.009 | 0.00146 |
| *p__Desulfobacterota* | 0.6597 | 0.03139 | 0.000852 |
| *p__Cyanobacteria* | 0 | 0.6816 | 0.00146 |

**Supplementary file 1b.** **The relative abundance of bacteria in CL and CON groups at genera level**

| Species name | CL-mean(%) | CON-mean(%) | P_value |
| --- | --- | --- | --- |
| *g__Bacteroides* | 25.7 | 9.822 | 0.01359 |
| *g__norank_f__Muribaculaceae* | 0 | 28.22 | 0.00041 |
| *g__Lactobacillus* | 11.53 | 3.751 | 0.02395 |
| *g__Escherichia-Shigella* | 11.81 | 2.311 | 0.01359 |
| *g__Helicobacter* | 7.648 | 4.838 | 0.8748 |
| *g__Faecalibaculum* | 10 | 1.242 | 0.005385 |
| *g__Ligilactobacillus* | 6.333 | 4.887 | 0.06608 |
| *g__Clostridium_sensu_stricto_1* | 9.278 | 0.9147 | 0.01779 |
| *g__unclassified_f__Lachnospiraceae* | 1.645 | 3.812 | 0.06608 |
| *g__Parasutterella* | 2.555 | 1.177 | 0.01813 |
| *g__Erysipelatoclostridium* | 1.127 | 2.219 | 0.0829 |
| *g__Prevotellaceae_UCG-001* | 0 | 3.331 | 0.00041 |
| *g__norank_f__norank_o__Clostridia_UCG-014* | 0.9409 | 2.364 | 0.2701 |
| *g__Lachnospiraceae_NK4A136_group* | 1.175 | 1.757 | 0.634 |
| *g__Akkermansia* | 0.007135 | 2.867 | 0.000682 |
| *g__Parabacteroides* | 1.38 | 1.15 | 1 |
| *g__Rikenellaceae_RC9_gut_group* | 0 | 2.486 | 0.00041 |
| *g__unclassified_o__Oscillospirales* | 0 | 2.376 | 0.03247 |
| *g__Alloprevotella* | 0 | 2.273 | 0.00041 |
| *g__Anaerotruncus* | 1.741 | 0.4362 | 0.4295 |
| *g__Muribaculum* | 0 | 2.142 | 0.00041 |
| *g__Dubosiella* | 0 | 2.092 | 0.00041 |
| *g__Bifidobacterium* | 0 | 1.967 | 0.00041 |
| *g__Clostridioides* | 1.172 | 0.7435 | 0.09847 |
| *g__Alistipes* | 0 | 1.508 | 0.00041 |
| *g__unclassified_c__Bacilli* | 0.5903 | 0.8662 | 0.3973 |
| *g__Mucispirillum* | 0 | 1.009 | 0.00146 |
| *g__Romboutsia* | 0.3582 | 0.4367 | 0.7929 |
| *g__uncultured_f__uncultured_o__Rhodospirillales* | 0.1689 | 0.6055 | 0.05834 |
| *g__unclassified_f__Ruminococcaceae* | 0.5584 | 0.157 | 0.08244 |
| *g__Bilophila* | 0.6597 | 0.03139 | 0.000852 |
| *g__norank_f__norank_o__Gastranaerophilales* | 0 | 0.6816 | 0.00146 |
| *g__Roseburia* | 0.478 | 0.1213 | 0.8663 |
| *g__Blautia* | 0.3701 | 0.1422 | 0.1409 |
| *g__unclassified_f__Oscillospiraceae* | 0.1779 | 0.2997 | 0.02354 |
| *g__uncultured_f__Oscillospiraceae* | 0.303 | 0.1717 | 0.2146 |
| *g__Eubacterium_siraeum_group* | 0.3743 | 0.07373 | 0.04048 |
| *g__Ruminococcus* | 0 | 0.3824 | 0.00041 |
| *g__norank_f__Eubacterium_coprostanoligenes_group* | 0.1037 | 0.2778 | 0.9163 |
| *g__unclassified_f__Atopobiaceae* | 0 | 0.3691 | 0.00041 |
| *g__norank_f__Ruminococcaceae* | 0.3648 | 0.001427 | 0.2143 |
| *g__Enterococcus* | 0.294 | 0.06421 | 0.003619 |
| *g__ASF356* | 0 | 0.3311 | 0.03247 |
| *g__Odoribacter* | 0 | 0.3196 | 0.00041 |
| *g__A2* | 0.2321 | 0.04091 | 0.5465 |
| *g__Lachnoclostridium* | 0.185 | 0.06326 | 0.263 |
| *g__Colidextribacter* | 0.03615 | 0.2121 | 0.03764 |
| *g__unclassified_f__Enterobacteriaceae* | 0.04804 | 0.1651 | 0.2497 |
| *g__Enterobacter* | 0.04233 | 0.1475 | 0.2172 |
| *g__Coprobacillus* | 0.1789 | 0 | 0.1709 |
| *g__Eubacterium_xylanophilum_group* | 0.04566 | 0.1241 | 0.1706 |
| *g__Ruminococcus_torques_group* | 0 | 0.1603 | 0.00041 |
| *g__Eubacterium_nodatum_group* | 0 | 0.1408 | 0.00041 |
| *g__Incertae_Sedis* | 0.1123 | 0.0195 | 0.007276 |
| *g__Proteus* | 0.1194 | 0.003805 | 0.000819 |
| *g__Enterorhabdus* | 0 | 0.1218 | 0.000405 |
| *g__Marvinbryantia* | 0 | 0.1132 | 0.00146 |
| *g__Oscillibacter* | 0.009513 | 0.1013 | 0.07475 |
| *g__unclassified_f__Erysipelatoclostridiaceae* | 0 | 0.1094 | 0.004569 |
| *g__UBA1819* | 0 | 0.1089 | 0.03225 |
| *g__uncultured_f__Erysipelotrichaceae* | 0 | 0.1061 | 0.001446 |

**Supplementary file 1c. The relative abundance of fungi in CL and CON groups at the phylum level**

| Species name | CL-mean(%) | CON-mean(%) | P_value |
| --- | --- | --- | --- |
| *p__Ascomycota* | 61.62 | 59.89 | 0.7929 |
| *p__unclassified_k__Fungi* | 21.24 | 33.73 | 0.04057 |
| *p__Basidiomycota* | 10.07 | 4.315 | 0.08312 |
| *p__Mucoromycota* | 7.059 | 1.948 | 0.2202 |
| *p__Chytridiomycota* | 0.007881 | 0.1182 | 0.1105 |

**Supplementary file 1d.** **The relative abundance of fungi in CL and CON groups at the genera level**

| Species name | CL-mean(%) | CON-mean(%) | P_value |
| --- | --- | --- | --- |
| *g__unclassified_k__Fungi* | 21.24 | 33.73 | 0.04057 |
| *g__unclassified_p__Ascomycota* | 7.986 | 28.35 | 0.007406 |
| *g__Aspergillus* | 17.96 | 9.871 | 0.03132 |
| *g__Microascus* | 4.087 | 3.386 | 0.188 |
| *g__Penicillium* | 5.256 | 2.012 | 0.0452 |
| *g__Mucor* | 5.364 | 0.442 | 0.4825 |
| *g__Candida* | 0 | 5.758 | 0.3816 |
| *g__Cladosporium* | 4.156 | 0.5539 | 0.001801 |
| *g__Trichoderma* | 3.138 | 1.366 | 0.1058 |
| *g__Wallemia* | 4.177 | 0.2673 | 0.000785 |
| *g__Claviceps* | 3.333 | 0 | 0.3816 |
| *g__Saccharomyces* | 1.437 | 1.678 | 0.9575 |
| *g__unclassified_f__Pleosporaceae* | 2.117 | 0.5674 | 0.6025 |
| *g__Apiotrichum* | 1.196 | 1.471 | 0.9039 |
| *g__Rhizopus* | 0.9233 | 1.506 | 0.8481 |
| *g__Malassezia* | 0.8814 | 0.8792 | 0.6025 |
| *g__unclassified_o__Microascales* | 1.423 | 0 | 0.1709 |
| *g__unclassified_o__Xylariales* | 1.234 | 0 | 0.3816 |
| *g__Thielaviopsis* | 0.8518 | 0.1605 | 0.3342 |
| *g__Meyerozyma* | 0.3146 | 0.6774 | 1 |
| *g__Letendraea* | 0 | 0.8723 | 0.3816 |
| *g__Sporobolomyces* | 0.4457 | 0.3931 | 0.945 |
| *g__Naganishia* | 0.4492 | 0.3121 | 0.7001 |
| *g__Alternaria* | 0.7509 | 0 | 0.03247 |
| *g__Hannaella* | 0.7484 | 0 | 0.3816 |
| *g__Trichosporon* | 0.7272 | 0 | 0.3816 |
| *g__Aureobasidium* | 0 | 0.7162 | 0.3816 |
| *g__Toxicocladosporium* | 0.7014 | 0 | 0.3816 |
| *g__Neosetophoma* | 0.6904 | 0 | 0.3816 |
| *g__unclassified_o__Sordariales* | 0 | 0.6188 | 0.3816 |
| *g__unclassified_c__Sordariomycetes* | 0.5737 | 0.005359 | 0.4881 |
| *g__Scopulariopsis* | 0.3237 | 0.214 | 1 |
| *g__Byssochlamys* | 0.5362 | 0 | 0.3816 |
| *g__Talaromyces* | 0 | 0.5283 | 0.3816 |
| *g__Simplicillium* | 0.1491 | 0.3647 | 1 |
| *g__Xeromyces* | 0.5069 | 0 | 0.1709 |
| *g__unclassified_f__Aspergillaceae* | 0.168 | 0.3253 | 0.3854 |
| *g__unclassified_f__Nectriaceae* | 0.3449 | 0.1204 | 0.4881 |
| *g__Chaetomium* | 0 | 0.4527 | 0.3816 |
| *g__unclassified_o__Mucorales* | 0.4196 | 0 | 0.3816 |
| *g__Megacapitula* | 0.4089 | 0 | 0.3816 |
| *g__Udeniomyces* | 0 | 0.3802 | 0.3816 |
| *g__Tilletia* | 0.3761 | 0 | 0.1709 |
| *g__unclassified_f__Microascaceae* | 0.36 | 0 | 0.1709 |
| *g__Yarrowia* | 0.3565 | 0 | 0.3816 |
| *g__Filobasidium* | 0.3417 | 0 | 0.3816 |
| *g__unclassified_o__Onygenales* | 0.2427 | 0.09331 | 1 |
| *g__Thielavia* | 0 | 0.3108 | 0.3816 |
| *g__Pichia* | 0 | 0.2976 | 0.3816 |
| *g__Fusicolla* | 0 | 0.2919 | 0.3816 |
| *g__Pseudogymnoascus* | 0.2894 | 0 | 0.07645 |
| *g__Pithoascus* | 0.2837 | 0 | 0.3816 |
| *g__Bulleromyces* | 0.2755 | 0 | 0.3816 |
| *g__Trematosphaeria* | 0.2702 | 0 | 0.3816 |
| *g__Coprinellus* | 0 | 0.2538 | 0.3816 |
| *g__Symmetrospora* | 0 | 0.2405 | 0.3816 |
| *g__Trichomerium* | 0.2298 | 0 | 0.3816 |
| *g__Kernia* | 0 | 0.215 | 0.3816 |
| *g__Xerochrysium* | 0.2147 | 0 | 0.3816 |
| *g__Rhizomucor* | 0.1917 | 0 | 0.1709 |
| *g__Starmerella* | 0.1857 | 0 | 0.3816 |
| *g__Nigrospora* | 0.1623 | 0 | 0.3816 |
| *g__Arthrinium* | 0.1617 | 0 | 0.3816 |
| *g__Acaulium* | 0.1526 | 0 | 0.3816 |
| *g__Fellomyces* | 0.1507 | 0 | 0.3816 |
| *g__Lichtheimia* | 0.1491 | 0.0009457 | 0.4881 |
| *g__Papiliotrema* | 0.1428 | 0 | 0.3816 |
| *g__Moesziomyces* | 0.1277 | 0 | 0.3816 |
| *g__unclassified_p__Chytridiomycota* | 0.007881 | 0.1182 | 0.1105 |
| *g__unclassified_o__Capnodiales* | 0.1198 | 0 | 0.3816 |
| *g__unclassified_f__Trichosporonaceae* | 0 | 0.1078 | 0.3816 |
