# Supplementary material for: Intestinal microbiome dysbiosis increases Mycobacteria pulmonary colonization in mice by regulating the Nos2-associated pathways
Source: eLife. 2024 Oct 16;13:RP99282. doi: 10.7554/eLife.99282 (PMC11483126; doi:10.7554/eLife.99282)
Supplement: Supplementary file 2. — (a) The primary enrichment pathways of differentially expressed genes (DEGs) in KEGG analysis at classification level 1. (b) The primary enrichment pathways of DEGs in KEGG analysis at classification level 2. (c) The primers of RT-qPCR. [file elife-99282-supp2.docx]

**Supplementary file 2. The KEGG enrichment pathways analysis of the lung transcriptome data and the primers of RT-qPCR.**

**Supplementary file 2a. The** **primary enrichment pathways of DEGs in KEGG analysis at classification level 1**

| Classification_level1 | Type | Gene_number | Percentage | Gene |
| --- | --- | --- | --- | --- |
| Organismal Systems | DEG | 3 | 6.98 | *Sema4d; Sirpb1c; Tnf* |
| Organismal Systems | DEG | 1 | 2.33 | *Hmox1* |
| Organismal Systems | DEG | 6 | 13.95 | *Ctsd; Nfkbie; Nos2; Slc26a4; Tnf; Trpm2* |
| Organismal Systems | DEG | 28 | 65.12 | *A2m; Ccl9; Cd14; Cd209a; Cd3e; Cd74; Cd80; Cfb; Clec4e; Cxcl1; Cxcl3; Cxcl5; Fcer2a; H2-M2; Icos; Il6; Lcn2; Marcksl1; Mefv; Naip2; Nfkbie; Nlrp1b; Nlrp3; Siglech; Sting1; Tbxas1; Tnf; Trpm2* |
| Organismal Systems | DEG | 3 | 6.98 | *Htr7; Nfkbie; Slc6a12* |
| Metabolism | DEG | 2 | 4.65 | *Arg1; Nos2* |
| Metabolism | DEG | 1 | 2.33 | *B4galnt4* |
| Metabolism | DEG | 2 | 4.65 | *Pla2g7; Tbxas1* |
| Metabolism | DEG | 1 | 2.33 | *Hmox1* |
| Human Diseases | DEG | 9 | 20.93 | *Bcl2a1b; Cd14; Cd3e; H2-M2; Hmox1; Il6; Nfkbie; Nos2; Tnf* |
| Human Diseases | DEG | 4 | 9.3 | *Bcl2a1b; Cd14; Hmox1; Nos2* |
| Human Diseases | DEG | 10 | 23.26 | *Cd14; Cd80; Ctsd; Cxcl1; Cxcl3; H2-M2; Hmox1; Il6; Nlrp3; Tnf* |
| Human Diseases | DEG | 2 | 4.65 | *Il6; Tnf* |
| Human Diseases | DEG | 4 | 9.3 | *Cd80; H2-M2; Il6; Tnf* |
| Human Diseases | DEG | 9 | 20.93 | *Cd3e; Cd80; Cxcl1; Cxcl3; Cxcl5; H2-M2; Icos; Il6; Tnf* |
| Human Diseases | DEG | 15 | 34.88 | *Cd14; Cd209a; Cd74; Cfb; Clec4e; Ctsd; Cxcl1; Cxcl3; Cxcl5; Il6; Mefv; Naip2; Nlrp3; Nos2; Tnf* |
| Human Diseases | DEG | 9 | 20.93 | *Arg1; Cd14; Cd3e; Cxcl1; Cxcl3; Il6; Marcksl1; Nos2; Tnf* |
| Human Diseases | DEG | 13 | 30.23 | *Cd209a; Cd3e; Cd74; Cfb; Cxcl1; Cxcl3; Fcer2a; H2-M2; Il6; Nfkbie; Nlrp3; Sting1; Tnf* |
| Human Diseases | DEG | 3 | 6.98 | *Il6; Nos2; Tnf* |
| Environmental Information Processing | DEG | 13 | 30.23 | *Bcl2a1b; Cd14; Ctsd; Cxcl1; Cxcl3; Cxcl5; Hmox1; Htr7; Il6; Inhba; Nos2; Timp1; Tnf* |
| Environmental Information Processing | DEG | 11 | 25.58 | *Ccl9; Cd80; Cxcl1; Cxcl3; Cxcl5; H2-M2; Htr7; Icos; Il6; Inhba; Tnf* |
| Cellular Processes | DEG | 8 | 18.6 | *Bcl2a1b; Ctsd; H2-M2; Hmox1; Il6; Nlrp3; Slc7a11; Tnf* |
| Cellular Processes | DEG | 1 | 2.33 | *Inhba* |
| Cellular Processes | DEG | 5 | 11.63 | *Cd14; Cd209a; Ctsd; H2-M2; Nos2* |

**Supplementary file 2b. The** **primary enrichment pathways of DEGs in KEGG analysis at classification level 2**

| Term | Classification_level2 | ListHits | geneID |
| --- | --- | --- | --- |
| Lipid and atherosclerosis | Cardiovascular disease | 6 | *Cd14;Cxcl1;Cxcl3;Il6;Nlrp3;Tnf* |
| Rheumatoid arthritis | Immune disease | 6 | *Cd80;Cxcl1;Cxcl3;Cxcl5;Il6;Tnf* |
| Graft-versus-host disease | Immune disease | 4 | *Cd80;H2-M2;Il6;Tnf* |
| NOD-like receptor signaling pathway | Immune system | 10 | *Cxcl1;Cxcl3;Il6;Mefv;Naip2;Nlrp1b;Nlrp3;Sting1;Tnf;Trpm2* |
| IL-17 signaling pathway | Immune system | 6 | *Cxcl1;Cxcl3;Cxcl5;Il6;Lcn2;Tnf* |
| Hematopoietic cell lineage | Immune system | 6 | *Cd14;Cd3e;Fcer2a;Il6;Siglech;Tnf* |
| C-type lectin receptor signaling pathway | Immune system | 5 | *Cd209a;Clec4e;Il6;Nlrp3;Tnf* |
| Intestinal immune network for IgA production | Immune system | 3 | *Cd80;Icos;Il6* |
| Toll-like receptor signaling pathway | Immune system | 4 | *Cd14;Cd80;Il6;Tnf* |
| T cell receptor signaling pathway | Immune system | 4 | *Cd3e;Icos;Nfkbie;Tnf* |
| Legionellosis | Infectious disease: bacterial | 6 | *Cd14;Cxcl1;Cxcl3;Il6;Naip2;Tnf* |
| Pertussis | Infectious disease: bacterial | 6 | *Cd14;Cxcl5;Il6;Nlrp3;Nos2;Tnf* |
| Tuberculosis | Infectious disease: bacterial | 8 | *Cd14;Cd209a;Cd74;Clec4e;Ctsd;Il6;Nos2;Tnf* |
| Amoebiasis | Infectious disease: parasitic | 7 | *Arg1;Cd14;Cxcl1;Cxcl3;Il6;Nos2;Tnf* |
| Chagas disease | Infectious disease: parasitic | 4 | *Cd3e;Il6;Nos2;Tnf* |
| Epstein-Barr virus infection | Infectious disease: viral | 6 | *Cd3e;Fcer2a;H2-M2;Il6;Nfkbie;Tnf* |
| NF-kappa B signaling pathway | Signal transduction | 5 | *Bcl2a1b;Cd14;Cxcl1;Cxcl3;Tnf* |
| TNF signaling pathway | Signal transduction | 5 | *Cxcl1;Cxcl3;Cxcl5;Il6;Tnf* |
| Viral protein interaction with cytokine and cytokine receptor | Signaling molecules and interaction | 6 | *Ccl9;Cxcl1;Cxcl3;Cxcl5;Il6;Tnf* |
| Cytokine-cytokine receptor interaction | Signaling molecules and interaction | 7 | *Ccl9;Cxcl1;Cxcl3;Cxcl5;Il6;Inhba;Tnf* |

**Supplementary file 2c. The primers of RT-qPCR**

| Amplified genes | Primer sequences (5' → 3') |
| --- | --- |
| *Cd74* | F: TAGACAAGCTGACCATCACCTCC |
|  | R: TGGGTCATGTTGCCGTACTTG |
| *Tnf* | F: CAAAATTCGAGTGACAAGCCTG |
|  | R: GAGATCCATGCCGTTGGC |
| *Lcn2* | F: TGGCCCTGAGTGTCATGTG |
|  | R: CTCTTGTAGCTCATAGATGGTGC |
| *Lrg1* | F: CAGATTCCTCATTCCCTCAG |
|  | R: CGTGTCAAAGCCAGATAAAC |
| *Ctsd* | F: GCTTCCGGTCTTTGACAACCT |
|  | R: CACCAAGCATTAGTTCTCCTCC |
| *Saa3* | F: AGAGAGGCTGTTCAGAAGTTCA |
|  | R: AGCAGGTCGGAAGTGGTTG |
| *Bpifa1* | F: TGCCTTTGGCTGTAAGCCC |
|  | R: AGAATTGCCTCCTCCAGACTTTA |
| *Scgb3a2* | F: ACTGCCCTTCTCATCAACCG |
|  | R: CAGTCCTGTCACCAGATGTTC |
| *Nos2* | F: GGAGCGAGTTGTGGATTGTC |
|  | R: TGAGGGCTTGGCTGAGTGAG |
| *Defb1* | F: ACTCTCTGCTTACTTTTGTCTG |
|  | R: GGTGCCTTGAATTTTGGT |
| *β-actin* | F: GCTTCTAGGCGGACTGTTACT |
| (animal) | R: GCCTTCACCGTTCCAGTTTTT |
| *β-actin* | F：GTCCACCGCAAATGCTTCTA |
| (human) | R：TGCTGTCACCTTCACCGTTC |
