# Supplementary material for: Intestinal microbiome dysbiosis increases Mycobacteria pulmonary colonization in mice by regulating the Nos2-associated pathways
Source: eLife. 2024 Oct 16;13:RP99282. doi: 10.7554/eLife.99282 (PMC11483126; doi:10.7554/eLife.99282)
Supplement: Peer review history

# Editor report: eLife Assessment

**DOI:** 10.7554/eLife.99282.3.sa0

**Authors:** Kiyoshi Takeda

**Affiliations:** Osaka University Japan

**Keywords:** Compelling, Incomplete, Important

This **important** study shows the effect of gut dysbiosis on the colonization of mycobacteria in the lung. The data with comprehensive analysis of gene expression profiles in the lung with dysbiotic mice is **compelling** and goes beyond the current state of the art. However, the mechanistic insight, where the lung epithelial cell line was used, and the experiments with Mtb infection are currently **incomplete**.

# Referee report: Reviewer #1 (Public review)

**DOI:** 10.7554/eLife.99282.3.sa1

Summary:

This work sought to demonstrate that gut microbiota dysbiosis may promote the colonization of mycobacteria, and they tried to prove that Nos2 down-regulation was a key mediator of such gut-lung pathogenesis transition.

Strengths:

They did large-scale analysis of RNAs in lungs to analyze the gene expression of mice upon gut dysbiosis in MS-infected mice. This might help provide overview of gene pathways and critical genes for lung pathology in gut dysbiosis. This data is somewhat useful and important for the TB field.

Weaknesses:

(1) They did not use wide-type Mtb strain (e.g. H37Rv) to develop mouse TB infection models, and this may lead to the failure for establishment of TB granuloma and other TB pathology icons.

(2) The usage of in vitro assays based on A542 to examine the regulation function of Nos2 expression on NO and ROS may not be enough. A542 is not the primary Mtb infection target in the lungs.

(3) They did not examine the lung pathology upon gut dysbiosis to examine the true significance of increased colonization of Mtb.

(4) Most of the studies are based on MS-infected mouse models with lack of clinical significance.

# Author comment: Author response

**DOI:** 10.7554/eLife.99282.3.sa2

**Authors:** Meiqing Han, Xia Wang, Lin Su, Shiqi Pan, Ningning Liu, Duan Li, Liang Liu, JunWei Cui, Huajie Zhao, Fan Yang

**Affiliations:** Xinxiang Medical University Xinxiang China; First Affiliated Hospital of Xinxiang Medical University Xinxiang China; Xinxiang Medical University Xinxiang China; Xinxiang Medical University Xinxiang China; Xinxiang Medical University Xinxiang China; Xinxiang Medical University Xinxiang China; Xinxiang Medical University Xinxiang China; First Affiliated Hospital of Xinxiang Medical University Xinxiang China; Xinxiang Medical University Xinxiang China; Xinxiang Medical University Xinxiang China

The following is the authors’ response to the original reviews.

**Reviewer #1 (Public Review):**

Summary:

This work sought to demonstrate that gut microbiota dysbiosis may promote the colonization of mycobacteria, and they tried to prove that Nos2 down-regulation was a key mediator of such gut-lung pathogenesis transition.

Strengths:

They did large-scale analysis of RNAs in lungs to analyze the gene expression of mice upon gut dysbiosis in MS-infected mice. This might help provide an overview of gene pathways and critical genes for lung pathology in gut dysbiosis. This data is somewhat useful and important for the TB field.

Weaknesses:

(1)They did not use wide-type Mtb strain (e.g. H37Rv) to develop mouse TB infection models, and this may lead to the failure of the establishment of TB granuloma and other TB pathology icons.

The colonization of *M.tb* in the lungs and the amount of colonization are the first and primary conditions for the occurrence of TB. Our aim in this study is to explore the impact of gut microbiota dysbiosis on the colonization of *M.tb* in the lungs. However, due to the lack of necessary conditions for biosafety in our laboratory, some highly infectious bacteria (such as *M.tb*) are not allowed to be cultured, and establishing the M.tb infection animal model in our laboratory does not meet the requirements of biosafety. Hence, we used the model strain of *M.tb*, M.smegmatis (MS), and established the animal-infected model for exploring the effect of gut microbiota dysbiosis on MS colonization in mice lungs. However, the establishment of MS infected model may not necessarily produce typical TB granulomas and other TB pathology signs. we have discussed the limitations of the current study in the discussion part of the manuscript. The suggested revisions are shown in lines 21-39 of page 15. In future studies, we plan to adopt the reviewer's suggestion and will use a wide-type *M.tb* strain to establish the TB-infected model in the laboratory that has biosafety standards to further verify the results of the current study.

(2) The usage of in vitro assays based on A542 to examine the regulation function of Nos2 expression on NO and ROS may not be enough. A542 is not the primary Mtb infection target in the lungs.

Thanks for the reviewer’s comments. Although alveolar epithelial cells (AECs) are not the main target cells of Mtb infection, they are among the cells that are contacted early in *M.tb* infection. Early *M.tb* invasion of AECs is very essential for the establishment of infection (PMID 11479618). AECs are usually the initial site of the lung’s response against *M.tb*. Available literature suggests that freshly isolated AECs are more permissive to *M.tb* growth than macrophages（PMID 33228849. As a cellular reservoir for *M.tb*, AECs are capable of facilitating rapid bacterial growth while potentially escaping recognition by phagocytes in the alveolus. The immune cells such as macrophages are the primary targets of *M.tb* infection, where the *M.tb* survive and proliferate, leading to the formation and maintenance of granulomas. However, AECs are subjected to the same density of infection, and the bacteria invade and replicate in these cells and induce cell apoptosis and necrosis, which is considered a major mechanism implicated in extra-pulmonary dissemination (PMID 12925134, PMID 32849525). Besides their direct barrier role, AECs also directly respond to *M.tb* infection by producing mediators such as cytokines, chemokines, and antimicrobial agents (PMID 35017314). Therefore, it is feasible to select alveolar epithelial cell A549 to explore the colonization mechanism of intestinal microbiota affecting *M.tb* in vitro.

(3) They did not examine the lung pathology upon gut dysbiosis to examine the true significance of increased colonization of Mtb.

We have added the results of the lung pathological section in the revised manuscript. The results of lung pathological sections are shown in lines 11-13 of page 4, and Figure S2 of supplement information.

(4) Most of the studies are based on MS-infected mouse models with a lack of clinical significance.

The first and primary condition of any pathogen infection is that the bacteria must invade the host through colonization and multiply in the target organ. This study aimed to investigate the effect of intestinal microbial dysbiosis on the colonization of mycobacterium in mouse lungs. Our laboratory does not meet the biosafety standard for culturing highly infectious bacteria such as *Mycobacterium tuberculosis*. So, we used the *Mycobacterium smegmatis* as a model strain for *M.tb* to establish the infected mice model in the current research. Although *M. smegmatis* is generally considered nonpathogenic, *M. smegmatis* is closely related to *M.tb* in biochemical characteristics, genetic information, cell structure, and metabolism (PMID 32674978). *M.smegmatis* is regarded as a valuable model organism in the study of *M.tb*, which is widely been used to explore the biological characteristics of *M.tb* such as physiological state, stress response, non-culture state reactivation, antimicrobial activity, and biochemical protection (PMID 32674978). It has also been reported that *M.smegmatis* could be used as a model strain to study the molecular mechanism of interaction between *M.tb* and its host (PMID 30546046, PMID25970481, PMID 29568875). However, in preclinical experimental research, we used *M. smegmatis* as the object of study. Instead of focusing on the pathological changes caused by *M.smegmatis* in the host lungs, we mainly focused on the influence of intestinal microbiota on the colonization of mycobacterium in the lungs and its possible mechanism, which provides a reliable model to study the prevention of early infection and spread of *M.tb* through regulating the intestinal microbiota. It has important clinical significance for the further development of new measures for the prevention and control of tuberculosis. If experimental conditions permit, the establishment of an infected model with wild-type *M.tb* can be used to verify the findings of the present study which may provide important clinical guidelines.

**Reviewer #2 (Public Review):**

The manuscript entitled "Intestinal microbiome dysbiosis increases Mycobacteria pulmonary colonization in mice by regulating the Nos2-associated pathways" by Han et al reported that using clindamycin, an antibiotic to selectively disorder anaerobic Bacteriodetes, intestinal microbiome dysbiosis resulted in Mycobacterium smegmatis (MS) colonization in the mice lungs. The authors found that clindamycin induced damage of the enterocytes and gut permeability and also enhanced the fermentation of cecum contents, which finally increased MS colonization in the mice's lungs. The study showed that gut microbiota dysbiosis up-regulated the Nos2 gene-associated pathways, leading to increased nitric oxide (NO) levels and decreased reactive oxygen species (ROS) and β-defensin 1 (Defb1) levels. These changes in the host's immune response created an antimicrobial and anti-inflammatory environment that favored MS colonization in the lungs. The findings suggest that gut microbiota dysbiosis can modulate the host's immune response and increase susceptibility to pulmonary infections by altering the expression of key genes and pathways involved in innate immunity. The authors reasonably provided experimental data and subsequent gene profiles to support their conclusion. Although the overall outcomes are convincing, there are several issues that need to be addressed:

(1) In Figure S1, the reviewer suggests checking the image sizes of the pathological sections of intestinal tissue from the control group and the CL-treatment group. When compared to the same intestinal tissue images in Figure S4, they do not appear to be consistently magnified at 40x. The numerical scale bars should be presented instead of just magnification such as "40x".

Thanks for the precise comments. We have carefully checked the pathological section in Figure S1 and Figure S5 and added the numerical scale bars to the figure. The revised sections are added in the supplementary materials.

(2) In Figure 4d, the ratio of Firmicutes in the CL-FMT group decreased compared to the CON-FMT group, whereas the CL-treatment group showed an increase in Firmicutes compared to the Control group in Figure 3b. The author should explain this discrepancy and discuss its potential implications on the study's findings.

The success of fecal microbial transfer (FMT) is influenced by many factors, such as host intestinal microbiota, immunity, and genetic factors (PMID 37167953). During FMT procedure, all microbiota of the donor feces do not have the same colonization ability in the recipients. Some research has revealed that the colonization success rate of *Bacteroidetes* is higher than that of *Firmicutes* [PMID 24637796]. In this study, we noticed that the reason for the difference between Figure 4D and Figure 3B was that during FMT, the colonization of Firmicutes decreased in the Cl-FMT receptor after transplantation, while the colonization of Bacteroides increased, resulting in a decrease in the proportion of *Firmicutes/ Bacteroides* in the Cl-FMT group. However, we considered the gut microbiota as a whole in the present study. After FMT, we found that 85.11% of bacterial genera and 52.38% of fungi genera present in the CL inocula were successfully transferred to the CL-recipient mice, and 91.45% of bacteria genera and 56.36% of fungi genera in the CON inocula were also successfully transferred to the CON-recipient mice, respectively (Figure 4g). The trans-kingdom network analyses between bacteria and fungi showed that the trends of the gut microbiome in recipient mice were consistent with those in the donor mice. Therefore, the FMT model established in this study remains successful. For reviewer clarification, we have added explanations in the discussion part of the manuscript. See lines 8-29 of page 12 for details.

(3) In Figure 6, did the authors have a specific reason for selecting Nos2 but not Tnf for further investigation? The expression level of the Tnf gene appears to be the most significant in both RT-qPCR and RNA-sequencing results in Figure 5f. Tnf is an important cytokine involved in immune responses to bacterial infections, so it is also a factor that can influence NO, ROS, and Defb1 levels.

Thanks for the valuable reviewer’s comment. By analyzing the transcriptome data, we found that there were 8 genes strongly associated with TB infection in the KEGG pathway, including *Nos2*, *Cd14*, *Tnf*, *Cd74*, *Clec4e*, *Ctsd*, *Cd209a,* and *Il6*. Then, we performed KO pathway analysis and found that the *Nos2* gene was strongly associated with multiple pathways including “cytokine activity ", "chemokine activity", and "nitric oxide synthase binding". Moreover, in a clinical study on tuberculosis, the expression level of *Nos2* in the plasma of patients with newly diagnosed tuberculosis was significantly higher than that of healthy people, indicating that *Nos2* is associated with the occurrence of tuberculosis (PMID 34847295). Therefore, we selected *Nos2* as the main target gene in the current study to conduct the correlation pathway analysis. As an important cytokine involved in the immune response to bacterial infection, *Tnf* mentioned by the reviewers may also be a factor affecting the levels of NO, ROS, and Defb1, which provides a new idea for our future research.

**Recommendations for the authors:**

**Reviewer #1 (Recommendations For The Authors):**

First, they need to use a true Mtb-infected mouse model to determine the relationship between gut dysbiosis and increased lung infection of Mtb.

Second, the mechanism by which nos2-mediated NO and ROS production need to be further analyzed in the real Mtb infection process (either in vivo or in vitro).

Third, Lung pathology should be included in addressing the increased colonization of mycobacteria. Addressing these problems may help improve this work.

(1) Our laboratory does not meet the biosafety standard for culturing highly infectious bacteria such as *Mycobacterium tuberculosis*. So, we used the *Mycobacterium smegmatis* as a model strain for *M.tb* to establish the infected mice model in the current research. Although *M. smegmatis* is generally considered nonpathogenic. *M. smegmatis* is closely related to *M.tb* in biochemical characteristics, genetic information, cell structure, and metabolism (PMID 32674978). *M.smegmatis* is regarded as a valuable model organism in the study of *M.tb*, and has been widely used to explore the biological characteristics of *M.tb* such as physiological state, stress response, non-culture state reactivation, antimicrobial activity, and biochemical protection (PMID 32674978). It has also been reported that *M.smegmatis* was used as a model strain to study the molecular mechanism of interaction between *M.tb* and its host (PMID 30546046, PMID25970481, PMID 29568875). However, in preclinical experimental research, we mainly focused on the influence of intestinal microbiota on the colonization of mycobacterium in the lungs and its possible mechanism which provides a reliable model to study the prevention of early infection and spread of *M.tb* through regulating the intestinal microbiota.

(2) In the future, we will establish an infected model with wild-type *M.tb* to verify the mechanism by which nos2-mediated NO and ROS production and promote M.tb colonization.

(3) We have added the results in the lung pathological section in the revised manuscript. The results of lung pathological sections are shown in lines 11-13 of page 4, and Figure S2 of supplement information.
